# Berberine Attenuates Chronic Atrophic Gastritis Induced by MNNG and Its Potential Mechanism

**DOI:** 10.3389/fphar.2021.644638

**Published:** 2021-03-25

**Authors:** Yuling Tong, Liping Liu, Ruilin Wang, Tao Yang, Jianxia Wen, Shizhang Wei, Manyi Jing, Wenjun Zou, Yanling Zhao

**Affiliations:** ^1^ College of Pharmacy, Chengdu University of Traditional Chinese Medicine, Chengdu, China; ^2^ Department of Pharmacy, Chinese PLA General Hospital, Beijing, China; ^3^ Integrative Medical Center, The Fifth Medical Center of PLA General Hospital, Beijing, China; ^4^ College of Clinical Medicine, Chengdu University of Traditional Chinese Medicine, Chengdu, China

**Keywords:** chronic atrophic gastritis, berberine, MNNG, TGF-β1, PI3K/AKT/mTOR

## Abstract

The purpose of this study was to investigate the therapeutic effect of berberine (BBR) on MNNG-induced chronic atrophic gastritis (CAG) and the possible mechanism of BBR through TGF-β1/PI3K signal pathway. GES-1 were pretreated with MNNG for 2 h before BBR treatment in all procedures. Cell viability was quantified by cell counting kit-8, and GES-1 morphology and proliferation were detected by high content screening (HCS) assay. The rat model of CAG was established by MNNG, and the therapeutic effect of BBR on stomach histopathology and serum supernatant were analyzed *in vivo*. In addition, the possible mechanism of BBR was further discussed, and the expression of related genes and proteins in TGF-β1/PI3K signal pathway was detected. The results showed that BBR could significantly improve the survival rate and morphological changes of GES-1, improve the gastric tissue injury of CAG rats, and reduce the expression of G-17 and inflammatory factors IL-8, TNF-α, IL-6 and IL-1β. In addition, BBR down-regulated the expression of TGF-β1 axis-related signals such as TGF-β1, PI3K, p-Akt/Akt, p-mTOR/mTOR and P70S6K, and promoted the expression of PTEN, LC3-II and Beclin-1. In Conclusion, BBR can improve CAG which may be closely related to TGF-β1/PI3K signal pathway.

## Introduction

Gastric cancer (GC) is the second leading cause of cancer-related death worldwide (Hallowell et al., 2019; Liou et al., 2020). Chronic atrophic gastritis (CAG) is a chronic gastric mucosal inflammation associated with the loss of gastric glandular cells, replaced by intestinal-type epithelium and fibrous tissue (Tian et al., 2019). The clinical symptoms of CAG are abdominal pain, abdominal discomfort, loss of appetite, weight loss and secondary anemia (Yang et al., 2018). As a precancerous lesion of gastric cancer, CAG plays an important role in the occurrence and development of gastric cancer (Weck et al., 2007; Thapa et al., 2019). Timely treatment of CAG can effectively prevent the occurrence of GC and has important clinical significance. Modern medicine mostly uses non-specific treatment for CAG, including eradication of *Helicobacter pylori*, use of antacid and mucosal protective agents (Tian et al., 2019). But the disease is easy to attack repeatedly and is difficult to cure. This not only seriously affects human health, but also puts a huge burden on the health care system (Chen et al., 2019), so there is an urgent need to find new and effective therapeutic drugs.

**FIGURE 1:**
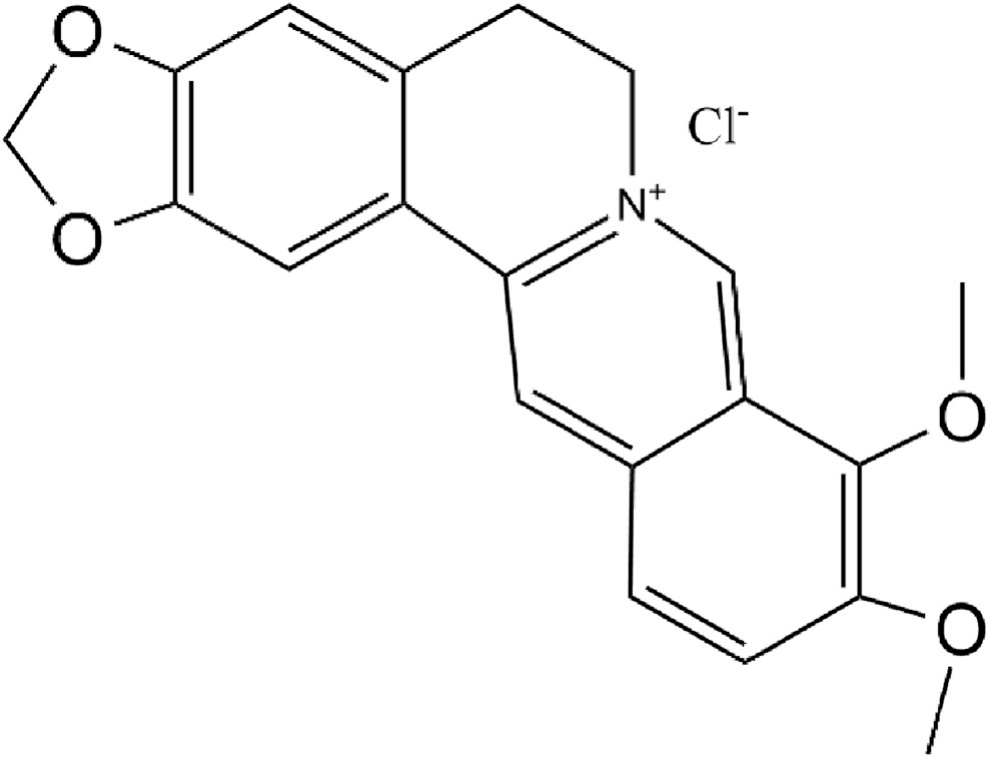
The chemical structure of berberine.

Berberine (BBR, Figure 1) is an isoquinoline alkaloid found in medicinal plants such as Coptis chinensis and Phellodendron Phellodendri. BBR has strong anti-inflammatory, antioxidant and immunomodulatory effects, so it is widely used in the treatment of chronic inflammation and gastrointestinal diseases (Wang et al., 2020). Berberine shows a good therapeutic effect on ulcerative colitis (Jing et al., 2020) and can improve glucose metabolism and insulin resistance by suppressing the inflammatory response (Shan et al., 2020). Recent studies have shown that BBR also has anti-tumor effects, such as promoting autophagy, anti-proliferation, apoptosis and so on (Liu J. et al., 2020; Samadi et al., 2020). However, the therapeutic effect of BBR on MNNG-induced CAG and its potential mechanism is still unclear.

Transforming growth factor-β (TGF-β) is a multifunctional cytokine, which can regulate various cellular functions such as cell proliferation, differentiation, migration and apoptosis, and plays an important role in maintaining immune homeostasis and preventing mucosal inflammation (Radaev et al., 2010). TGF-β and its signal pathway are mainly involved in the regulation of gastric mucosal inflammation (Nguyen et al., 2015), which may be the pathogenesis of many related diseases, including chronic inflammatory diseases and gastric cancer (Hong et al., 2010). Studies have shown that G3BP1 plays a role in promoting gastric cancer through TGF-β/smad signal pathway (Xiong et al., 2019). TGF-β1 can activate TGFβ1/smad2/3 classical signal pathway and non-classical PI3K/AKT/mTOR signal pathway. PI3K/Akt signal axis is activated during the occurrence and development of gastritis (Sokolova et al., 2014). Activated AKT can phosphorylate downstream substrates and promote cell proliferation (Xie and Liu, 2018). Studies have suggested that the occurrence of CAG is due to the destruction of the dynamic balance between gastric mucosal cell proliferation and apoptosis, the proliferation of malignant cells and the decrease of apoptosis (Rosania et al., 2017). Therefore, this study will explore the therapeutic effect of BBR on CAG and its possible mechanism through TGF-β1/PI3K/Akt signal axis, and lay a foundation for further investigate of the protective mechanism of BBR on CAG.

## Materials and Methods

### Ethics Statement

This study is conducted in strict accordance with the recommendations of the Guidelines for the Care and Use of Laboratory Animals of the Ministry of Science and Technology of China. All breeding and experiments are examined and approved by the Animal Ethics and Experimental Committee of the Fifth Medical Center of the PLA General Hospital (Approval ID: IACUC-2018-010).

### Drugs and Chemicals

BBR Standard (purity ≥ 98%, Cat. No. CHB181028) and 1-Methyl-3-nitro-1-nitrosoguanidine (MNNG, purity ≥ 98%, CAS. No. 70-25-7) were purchased from Chroma Biotechnology Co. Ltd. (Chengdu, China). BBR and MNNG were dissolved in dimethyl sulfoxide (DMSO) solution and diluted to the corresponding concentration when applied for human gastric epithelial cells (GES-1). BBR (purity ≥ 95%, Cat. No. CHB180606) was dissolved in sodium chloride injection (NS 0.9%) and diluted to the corresponding concentration when applied to rats. MNNG (purity 95%, Cat. No. K1922100) was purchased from Aladdin Biochemical Technology Co. Ltd. (Shanghai, China). MNNG dissolved in pure water and diluted to the corresponding concentration when applied to rats.

### Cell Culture and Cell Viability Assay

GES-1 lines were purchased from Fuheng Cell Center (Shanghai, China) and cultured in Dulbecco’s modified Eagle’s medium (DMEM) containing 10% fetal bovine serum (FBS), 100 IU/ml penicillin and 100 μg/ml streptomycin and cultured in 37°C cell incubator containing 5% CO_2_. GES-1 cells in logarithmic phase were treated with different concentrations of MNNG (12.5, 25, 30, 40, 50, 60, 80, 80, 100 μΜ) in the dark for 24 h. According to previous experiments (Yang et al., 2020), the GES-1 co-cultured with MNNG (40 μM) was treated with 20 and 40 μM BBR. 10% (vol/vol) cell counting kit-8 (CCK-8; Lot. PR829, Dojindo, Tokyo, Japan) was added into cells and incubated for 15 min at 37°C. The absorbance was measured at 450 nm. Cell viability was calculated according to the cell viability (%) = (OD treatment/OD control) × 100.

### Morphological Identification and Quantitative Statistics

Scan using Cell Health Profiling Assay module in High Content Screening System (Thermo Scientific, MA, United States). The morphology of GES-1 cells was detected by Fluorescent dyes, including Hechst33342 (H3570, Invitrogen), Calcein AM (BMD00064, Abbkine), and Ethidium homodimer-1 (EthD-1) (BMD00060, Abbkine). The parameters and forma setting were reported previously by O'Brien, et al. (O'Brien et al., 2006) and the wave lengths in different channels were set to collect fluorescent images. Finally, the mean fluorescence intensity of GES-1 cells was quantified by using an Array Scan XTI system.

### Animals

Specific pathogen free (SPF) male Sprague Dawley rats (150–170 g) purchased from Sibeife Animal Breeding Center (Beijing, China) (Permission No. SCXK-(jing) 2019-0010). All animals were adapted for at least one week before experiments and maintained in the SPF condition of temperature (25 ± 0.5°C), relative humidity (55 ± 5%), an alternating lighting (12 h light: 12 h dark cycle), and free access to sufficient food and water. The animals were randomly divided into four groups (*n* = 8), including control group, CAG model group, BBR low dose group (14 mg/kg), BBR high dose group (28 mg/kg)). The control group had free access to drinking water and food, and the other groups were given MNNG (170 μg/ml) free drinking combined with irregular diet (one day full feeding, one day fasting, and so repeatedly), and MNNG (170 μg/ml, 1 ml/100 g) was given intragastric administration every other day for 10 weeks (Zhu et al., 2013; Zhang and Wang, 2019). After the model was established, the corresponding drugs were given by gavage for four weeks (1 ml/100 g, once a day). After the last administration, rats were sacrificed under 20% ethyl carbamate solution. Also, blood samples and stomach tissues were collected.

### Histopathological Examinations

Stomach tissue samples were fixed and preserved in 10% neutral formalin solution. Then the sample was dehydrated in ethanol and xylene respectively. After dehydration, the tissue sample was embedded in paraffin wax. 5 μm thick serial slices were obtained by microtome and stained with hematoxylin-eosin (HE). Histopathology of stomach mucosal injury was determined by photography with light microscopy and 200×, 400× magnification was used in the microscopy analysis.

### Enzyme-Linked Immunosorbent Assays

The serum concentrations of gastrin 17 (G-17) (ml059375), interleukin 8 (IL-8) (ml037351) and tumor necrosis factor α (TNF-α) (ml002859) in rats were determined according to the instructions of the manufacturer in the Elisa kit (MLBIO, Shanghai, China). The concentration of ligand in serum was calculated according to the ng/L of ligand.

### Real-Time Quantitative PCR for mRNA Expression

The total RNA of control and MNNG-treated rats or cells were extracted with TRIzol reagent (Nordic Bioscience Co., Beijing, China) and were transformed into cDNA by reverse transcription kit (Thermo Fisher Scientific, United States) according to the manufacturer’s protocol. RT-PCR was performed on a QuantStudio™ Real-Time PCR System version 1.3 (Applied Biosystems by Thermo Fisher Scientific). Taking β-actin as the endogenous reference, the relative amount of mRNA is determined based on 2^−ΔΔCT^ calculation. The primer sequences of TGF-β1, PI3K, AKT, mTOR, PTEN, P70S6K, IL-6, IL-1β, LC3-II and beclin-1 were listed in Table 1.

**TABLE 1:** Primers sequences of real-time PCR analyses for mRNA expression.

Genes	Forward	Reverse
rats	—	—
TGF-β1	GAC​CGC​AAC​AAC​GCA​ATC​TAT​GAC	CTG​GCA​CTG​CTT​CCC​GAA​TGT​C
PI3K	GCT​GTT​GAT​AGA​CCA​CCG​CTT​CC	TGC​CCT​GTT​CCT​CTG​CCT​TCC
PTEN	TTG​AAG​ACC​ATA​ACC​CAC​CAC​AGC	CAT​TAC​ACC​AGT​CCG​TCC​TTT​CCC
P70S6K	TTC​AGC​CAG​CAC​AGC​AAA​TCC​TC	CCG​CTC​GTT​GTC​ACA​TCC​ATC​TG
IL-6	ACT​TCC​AGC​CAG​TTG​CCT​TCT​TG	TGG​TCT​GTT​GTG​GGT​GGT​ATC​CTC
IL-1β	CTC​ACA​GCA​GCA​TCT​CGA​CAA​GAG	TCC​ACG​GGC​AAG​ACA​TAG​GTA​GC
β-actin	CAC​TAT​CGG​CAA​TGA​GCG​GTT​CC	ACT​GTG​TTG​GCA​TAG​AGG​TCT​TTA​CG
—	—	—
GES-1	—	—
TGF-β	AGC​AAC​AAT​TCC​TGG​CGA​TAC​CTC	TCA​ACC​ACT​GCC​GCA​CAA​CTC
PI3K	CTT​TGC​GAC​AAG​ACT​GCC​GAG​AG	CGC​CTG​AAG​CTG​AGC​AAC​ATC​C
AKT	GCA​GGA​TGT​GGA​CCA​ACG​TGA​G	GCA​GGC​AGC​GGA​TGA​TGA​AGG
mTOR	CTT​GCT​GAA​CTG​GAG​GCT​GAT​GG	CCG​TTT​TCT​TAT​GGG​CTG​GCT​CTC
Beclin-1	ACA​TCT​GGC​ACA​GTG​GAC​AGT​TTG	AGC​ATG​GAG​CAG​CAA​CAC​AGT​C
LC3-II	GTC​AGC​GTC​TCC​ACA​CCA​ATC​TC	TCC​TGG​GAG​GCA​TAG​ACC​ATG​TAC
β-actin	AGG​AAG​GAC​CTG​TAT​GCC​AAC​A	GCG​CGG​TGA​TCT​CTT​TCT​G

### Western Blotting Assay

The total protein in the treated gastric tissue was extracted with the high-efficiency RIPA tissue/cell rapid lysate containing 1 mmol/L PMSF (Solarbio, Beijing, China) and the phosphatase inhibitor Cocktail III (TOPSCIENCE, Shanghai, China), and the BCA Protein Assay Kit (Solarbio, Beijing, China) was used for quantification. Protein bands were separated by SDS-PAGE and then transferred to polyvinylidene fluoride (PVDF) membrane (Millipore, MA, United States). Sealed in Tris-buﬀered saline (TBS) containing 5% bovine serum albumin (BSA) (Biotopped, Beijing, China) at room temperature for 1 h, then incubated with primary antibodies at 4°C overnight (Table 2). Then wash with TBS-0.1% Tween 20 (TBST) 3 times for 5 min each time, and incubate with horseradish peroxidase conjugated second antibody (goat anti-rabbit IgG) at room temperature for 1 h. Visualization of immunoblotting by enhanced chemiluminescence. GAPDH as internal reference. Quantitative analysis using Image-pro Plus 6.0 software.

**TABLE 2:** Antibodies.

Antibodies	Dilution	Manufacturers	Cat. no
Rabbit anti-TGF beta 1	1:500	Bioss	Bs-0086R
Rabbit anti-PI3 kinase p110 beta	1:500	Bioss	Bs-10657R
Rabbit anti-AKT	1:500	Bioss	Bs-0115R
Phospho-AKT (Ser473)	1:500	Affinity	AF0016
Rabbit anti-mTOR	1:500	Bioss	Bs-1992R
Phospho-mTOR (Ser2448)	1:1,000	Cell signaling technology	5536T
GAPDH monoclonal antibody	1:50,000	Proteintech	60004-1-Ig
Goat anti-rabbit IgG (H + L)	1:20,000	ZSGB-BIO	ZB-2301

### Statistics Analysis

All data were presented as mean ± standard deviation (SD) and analyzed with the SPSS software program (version 17.0; SPSS Inc., Chicago, IL, United States). Data were presented using one-way analysis of variance (ANOVA) followed by Bonferroni method. *p* < 0.05 was considered statistically significant and *p* < 0.01 was highly significant. GraphPad Prism software for Windows (version 6.02; Inc., San Diego, CA, United States) were utilized for visible presentation of all results.

## Results

### Cell Viability

**FIGURE 2:**
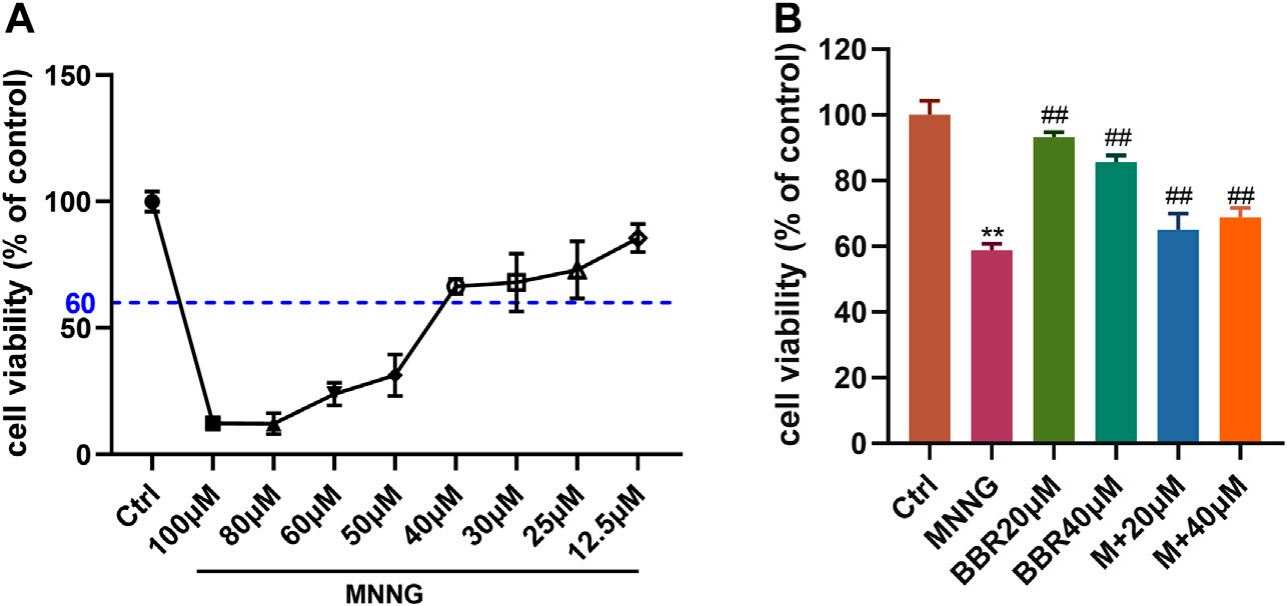
Eﬀect of MNNG and BBR on cell viability of GES-1 cells **(A)** Cell survival rate of GES-1 cells treated with different doses of MNNG for 24 h **(B)** Protective effect of berberine pretreatment on proliferation of MNNG co-cultured GES-1 cells. Data were shown as mean ± SD (*N* = 5). **<0.01 vs. control group. ##<0.01 vs. MNNG group. Ctrl, Control; M, MNNG.

The viability of GES-1 cells was detected by CCK-8 kit. First of all, the best concentration of MNNG (12.5, 25, 30, 40, 50, 60, 100 μM) on GES-1 cells was explored, and the best cell survival rate was more than 60%. The results showed that 40 μM MNNG had the best effect (Figure 2A). Then we explored the protective effect of 20 and 40 μM BBR on GES-1 co-cultured with MNNG (40 μM). The results showed that compared with MNNG group, the cell survival rate of 20 and 40 μM BBR co-culture with MNNG groups increased significantly (*p* < 0.01) (Figure 2B).

### GES-1 Morphological Identification and Quantitative Statistics

**FIGURE 3:**
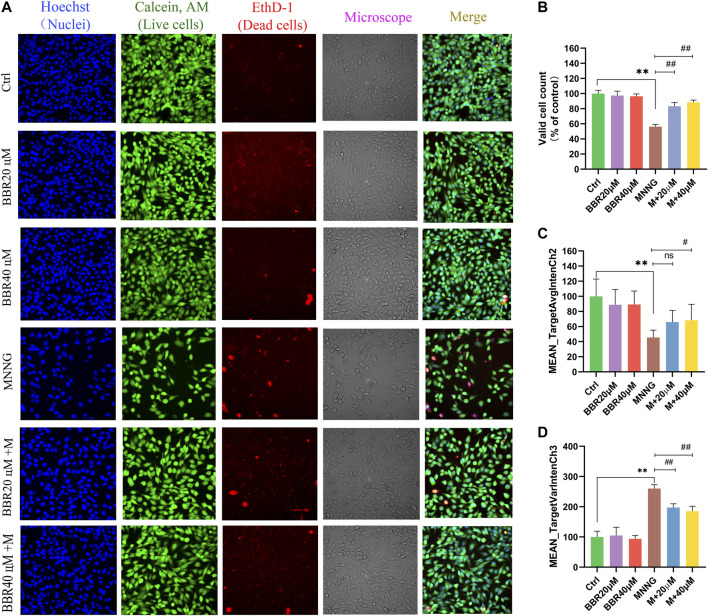
Morphological identification and quantitative analysis of HCS imaging assay for GES-1 cells **(A)** Representative microphotographs of HCS image analysis of GES-1 cells. Intensity of fluorescence staining reflected the survival cells (green fluorescence) and dead cells (red fluorescence) **(B)** Valid cell counts of GES-1 cells (% of control) **(C)** Live cell counts of GES-1 cells (MEAN_TargetAvgIntenCh2) **(D)** Dead cell counts of GES-1 cells (MEAN_TargetAvgIntenCh3). Data were shown as mean ± SD. **<0.01 vs. control group. ##<0.01 vs. MNNG group. Ns none-significant. Ctrl, Control; M, MNNG.

In order to more directly reflect the effect of BBR on cell morphology, we used high-content live-cell imaging assays to qualitatively and quantitatively assay cell count, cell morphology and cell viability of GES-1. As shown in Figure 3A, GES-1 in the control group, BBR 20 μΜ and BBR 40 μΜ groups presented Hoechst and Calcein AM fluorescence in the nuclear and cytoplasmic area. Compared with the control group, the morphology of cells changed after MNNG treatment. However, BBR treatment can alleviate this change in GES-1, especially at 40 μΜ. Cell count results show (Figure 3B) that the number of cells treated with MNNG was significantly lower than that of the control group (*p* < 0 01), and the decrease rate of cell number after BBR treatment was significantly lower than that of the MNNG group (*p* < 0 01). Compared with the control group, the green fluorescence was significantly decreased (*p* < 0.01) and the red fluorescence was significantly enhanced (*p* < 0.01) in the MNNG group, suggesting that the number of living cells decreased and the number of dead cells increased (Figures 3C,D). After BBR treatment, the green fluorescence of GES-1 was significantly enhanced and the red fluorescence was weakened (*p* < 0.05 or *p* < 0.01), and the effect of BBR40 fluorescence was more obvious. The results suggest that BBR can significantly improve the morphology and proliferation of GES-1 cells injured by MNNG.

### Effect of Berberine on Chronic Atrophic Gastritis Induced by MNNG

**FIGURE 4:**
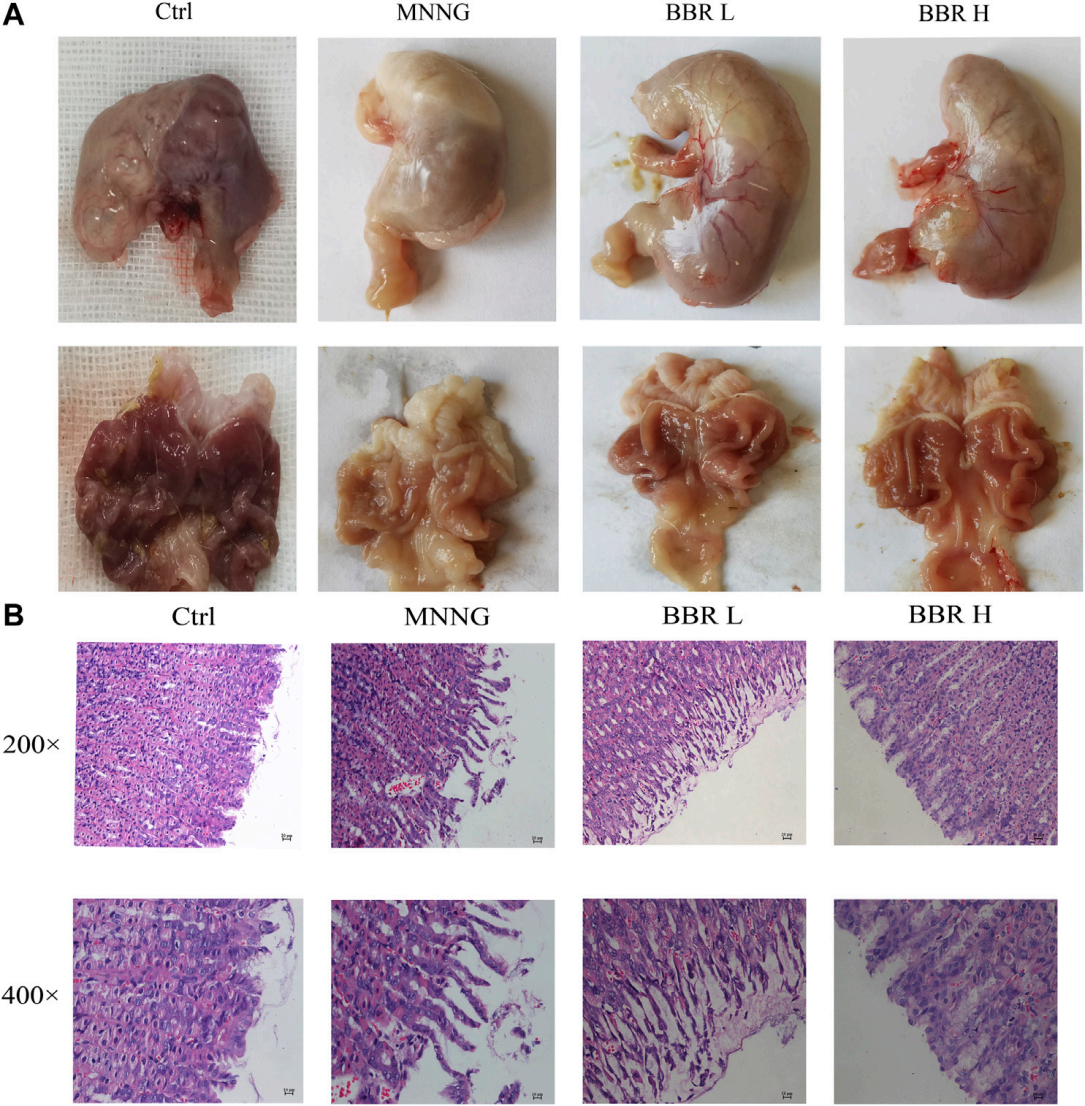
Effect of BBR on stomach histopathological changes **(A)** Representative morphology of stomach image of rats **(B)** Representative photomicrographs and summary data for HE staining of stomach histological changes were represented. Ctrl, Control; BBR L, BBR low dose group; BBR H, BBR high dose group.

In order to investigate the therapeutic effect of BBR on CAG induced by MNNG, we carried out an experiment *in vivo*. As can be seen from Figure 4A, compared with the control group, the color of gastric tissue in MNNG group became pallor and gastric folds became lighter. The pathological results showed that (Figure 4B), MNNG combined with irregular diet induced necrosis and exfoliation of gastric mucosal epithelial cells, atrophy and decrease of mucosal intrinsic glands, irregular distribution of residual intrinsic glands and infiltration of inflammatory cells in CAG rats. Compared with the MNNG group, the morphology of glands in the BBR group was more complete, the arrangement was regular, and the infiltration of inflammatory cells was significantly reduced, and the effect was more obvious in the high dose group. The results suggest that BBR, especially high dose, has a certain therapeutic effect on CAG induced by MNNG.

### Expression of Biomarkers in Serum

**FIGURE 5:**
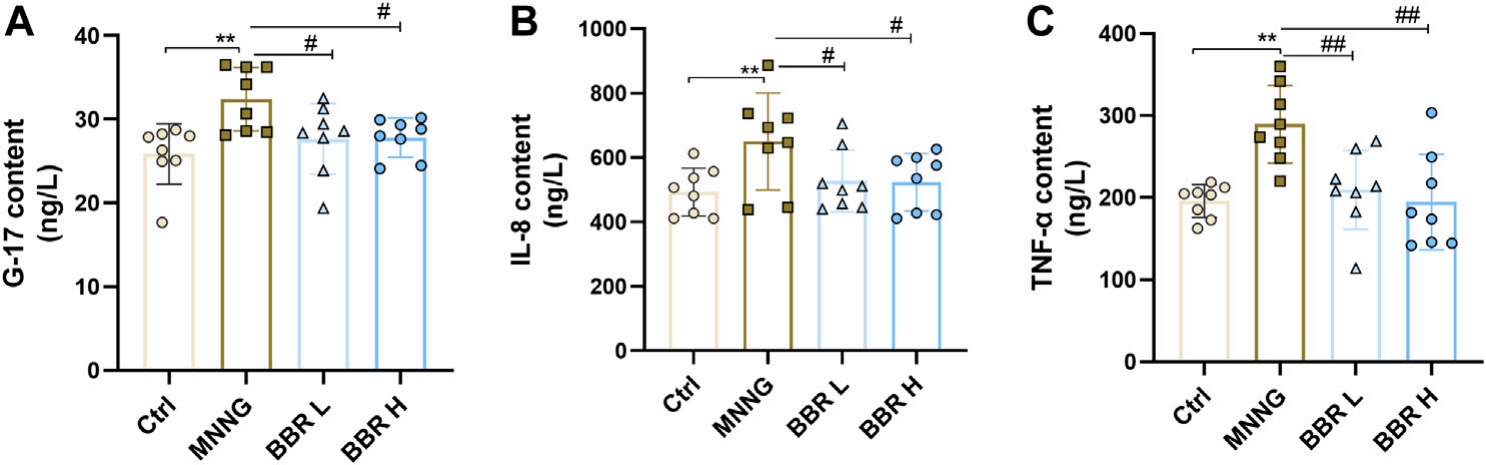
Detection of G-17, IL-8 and TNF-α expression in serum of CAG rats by ELISA kit. Data were shown as mean ± SD (*N* = 8). **<0.01 vs. control group. ##<0.01 vs. MNNG group; #<0.05 vs. MNNG group. Ctrl, Control; BBR L, BBR low dose group; BBR H, BBR high dose group.

In order to determine the biological activity of several specific serum markers induced by MNNG in CAG rats, the expression levels of G-17, IL-8 and TNF-α in serum supernatant were determined. As shown in Figure 5, the serum levels of G-17, IL-8 and TNF-α in CAG rats induced by MNNG were significantly increased than in the control group (*p* < 0.01). After administration of BBR (14and 28 mg/kg), the expression of G-17, IL-8 and TNF-a were significantly decreased (*p* < 0.01 or *p* < 0.05). These results suggest that BBR intervention can inhibit the activity of specific markers such as G17, IL-8 and TNF- a in CAG rats treated with MNNG.

### Effect of Berberine on GES-1 Gene Expression

**FIGURE 6:**
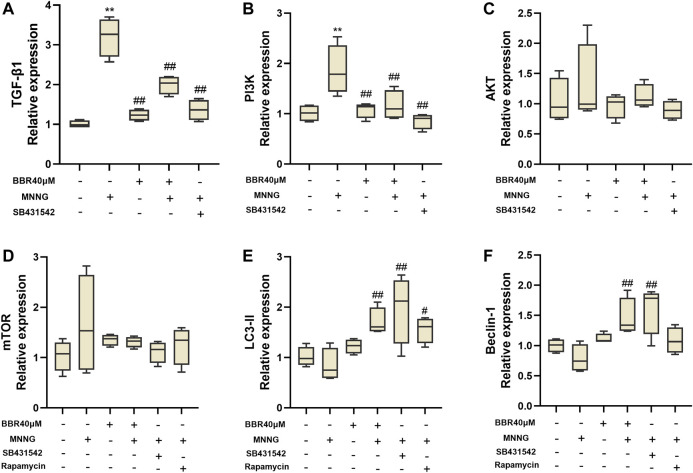
Histogram of TGF- β1 **(A)**, PI3K **(B)**, Akt **(C)**, mTOR **(D)**, LC3-II **(E)** and Beclin-1 **(F)** mRNA expression in MNNG co-cultured GES-1 cells by RT-PCR. Use SB-431542 and rapamycin to inhibit TGF- β1 and mTOR respectively. Data were shown as mean ± SD (*N* = 4). **<0.01 vs. control group; *<0.05 vs. control group. ##<0.01 vs. MNNG group; #<0.05 vs. MNNG group.

Firstly, the effect of BBR treatment on the related genes of TGF-β1/PI3K/Akt signal pathway in MNNG co-cultured GES-1 cells was verified *in vitro*. SB-431542 (Cat No. HY-10431, MedChem Express, Shanghai, China, 10 μΜ) (Liu X. S. et al., 2019), a small molecular inhibitor, was used to inhibit the expression of TGF-β1, and Rapamycin (Cat No. HY-10219,MedChem Express, Shanghai, China, 100 nΜ) (Liu X. et al., 2020) was used to inhibit the expression of mTOR. As shown in Figure 6A, the level of TGF-β1 in MNNG group increased significantly than control group. The level of TGF-β1 decreased significantly after using SB-431542 (*p* < 0.01). And after treatment with BBR, the TGF-β1 expression is also significantly decreased. In addition, the downstream index PI3K increased significantly after MMNG co-culture (*p* < 0.01), and decreased significantly after SB-431542 and BBR treatment (*p* < 0.01) (Figure 6B). Expression of Akt and mTOR in the MNNG group increased compared with the control group, but there was no significant difference (*p* > 0.05) (Figures 6C,D). The expression of LC3-II and Beclin-1, the downstream indexes of mTOR, increased significantly after treatment with Rapamycin and BBR (*p* < 0.01 or *p* < 0.05) (Figures 6E,F). These results suggest that the improvement of GES-1 damage induced by MNNG by BBR may be related to TGF-β1/Akt signal pathway.

### Effect of Berberine on Gene Expression in Chronic Atrophic Gastritis Rats

**FIGURE 7:**
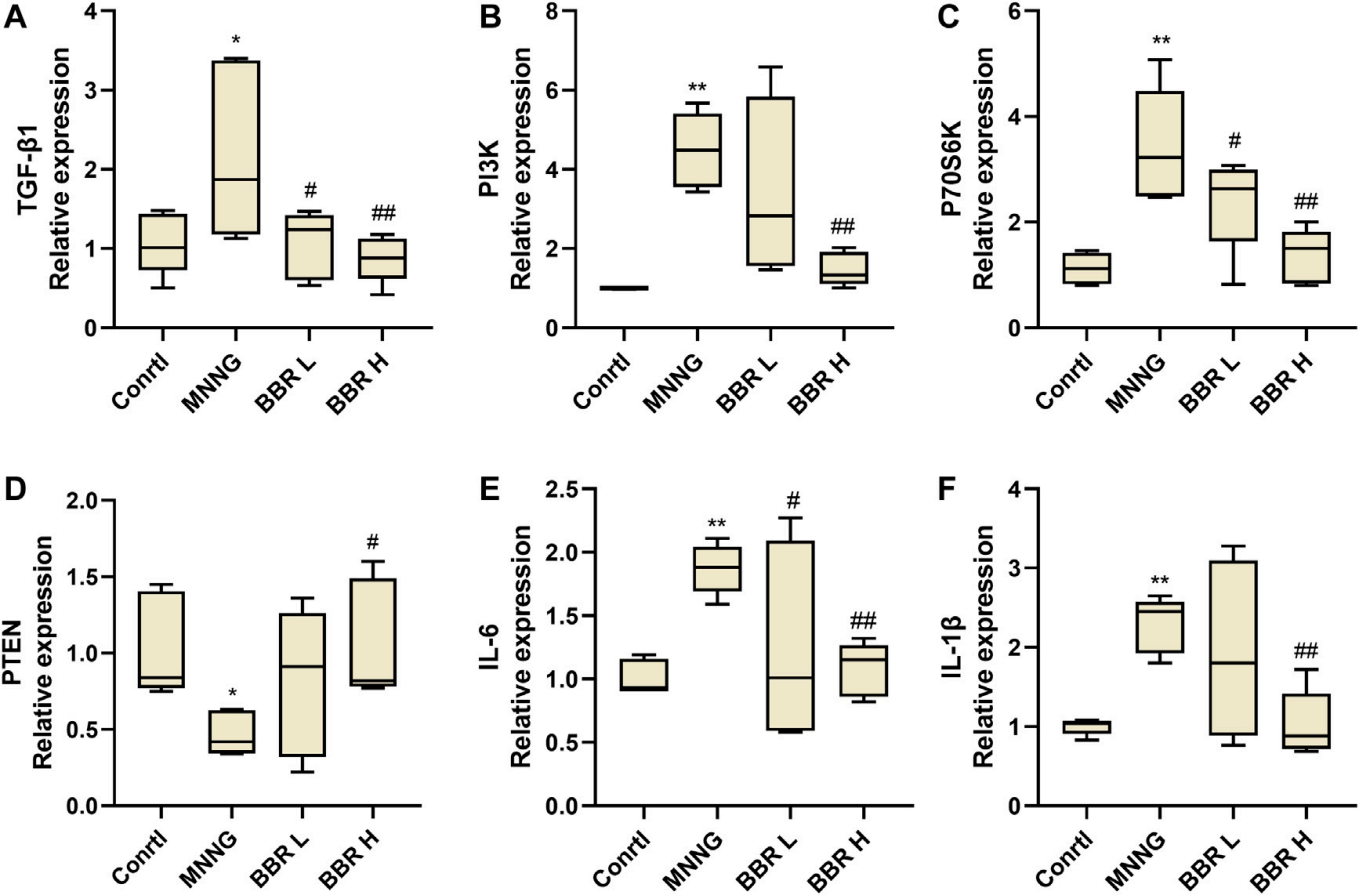
Histogram of TGF- β1 **(A)**, PI3K **(B)**, P70S6K **(C)**, PTEN **(D)**, IL-6 **(E)** and IL-1β **(F)** mRNA expression in gastric tissue of CAG rats by RT-PCR. Data were shown as mean ± SD (*N* = 5). **<0.01 vs. control group. ##<0.01 vs. MNNG group; #<0.05 vs. MNNG group. Ctrl, Control; BBR L, BBR low dose group; BBR H, BBR high dose group.

We further verified the effect of BBR on the expression of TGF-β1 and its downstream genes in gastric mucosa of CAG rats by RT-PCR. As shown in Figure 7, the expression of TGF-β1, PI3K and P70S6k was significantly increased in MNNG group (*p* < 0.01). After BBR (14and 28 mg/kg) administration, the levels were significantly decreased (*p* < 0.01 or *p* < 0.05). And compared with MNNG group, the expression of PTEN was significantly decreased in BBR high dose group (*p* < 0.05). In addition, the expression of inflammatory cytokines IL-6 and IL-1β in gastric mucosa of CAG rats was also measured (Figures 7E,F). The results showed that the expression of IL-8 and IL-1β in BBR groups were significantly decreased (*p* < 0.01 or *p* < 0.05).

### Berberine Inhibited Protein Expression in TGF-β1 Signaling Axis When MNNG Treatment

**FIGURE 8:**
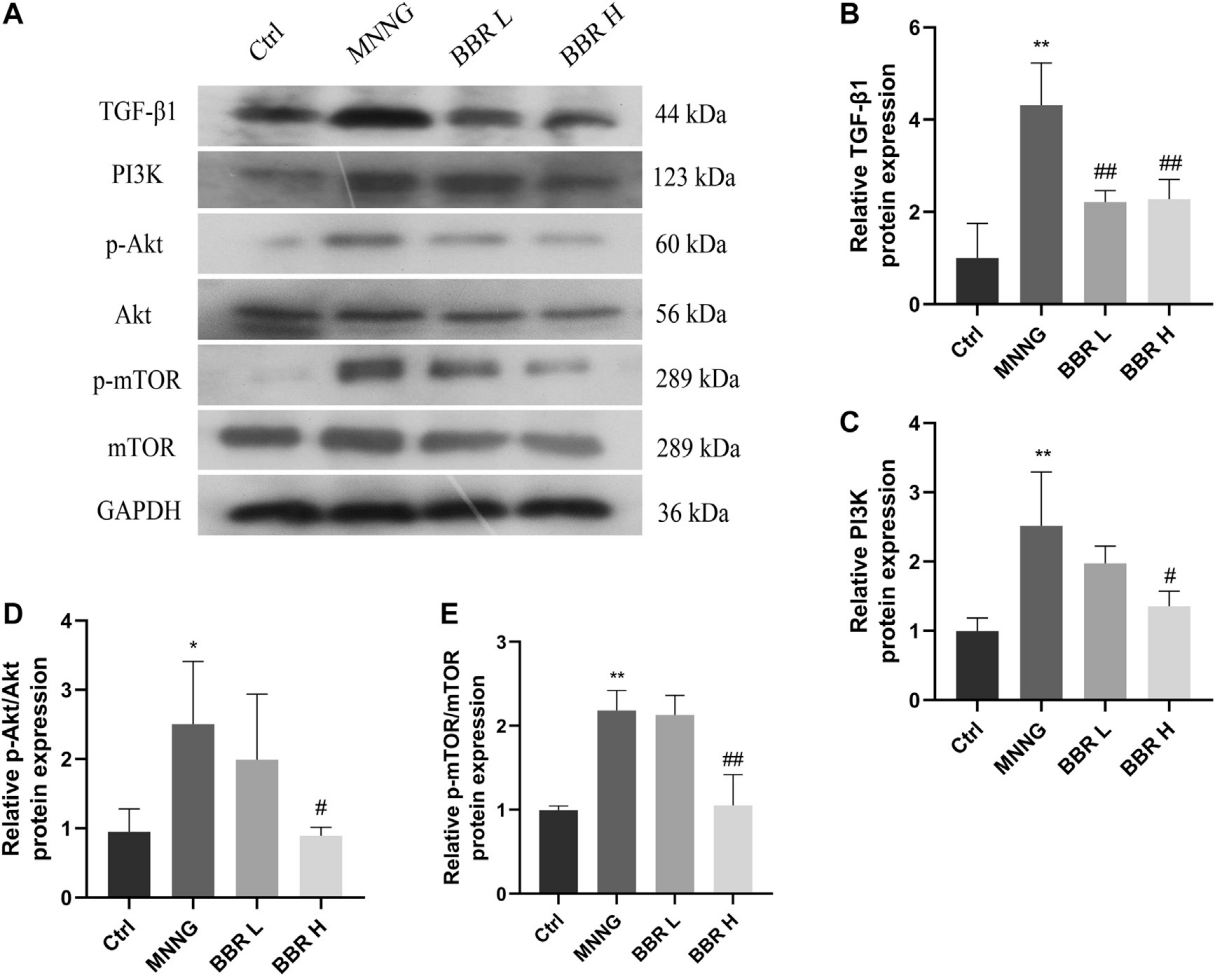
Effect of BBR on proteins expression in CAG rats **(A)** Western blotting images of TGF-β1, PI3K, p-Akt, Akt, p-mTOR, mTOR **(B)** Relative TGF-β1 protein level in stomach **(C)** Relative PI3K protein level in stomach **(D)** Relative p-Akt/Akt protein level in stomach **(E)** Relative p-mTOR/mTOR protein level in stomach. Data were shown as mean ± SD (*N* = 3). **<0.01 vs. control group; *<0.05 vs. control group. ##<0.01 vs. MNNG group; #<0.05 vs. MNNG group. Ctrl, Control; BBR L, BBR low dose group; BBR H, BBR high dose group.

We have previously proved that BBR can inhibit the mRNA expression of TGF-β1 and PI3K and their downstream related genes. Therefore, we detected the expression of related proteins on the axis of TGF-β1/PI3K/Akt signal. As shown in Figure 8, compared with the control group, the expression levels of TGF-β1, PI3K and p-Akt/Akt,p-mTOR/mTOR in MNNG group were significantly higher (*p* < 0.01 or *p* < 0.05). However, the high dose of BBR could significantly inhibit the expression of these proteins (*p* < 0.01 or *p* < 0.05). The above results further confirmed that the therapeutic effect of berberine on CAG maybe exerted through the TGF-β1/PI3K/Akt signal axis.

## Discussion

CAG is one of the precancerous stages of intestinal type gastric cancer (GC), which has a high prevalence rate and is still a serious medical problem troubling people all over the world (Liu H. et al., 2019). MNNG is a commonly used chemical carcinogen of stomach in recent years. MNNG simulates improper intake of nitrate (such as pickled foods), leading to precancerous lesions such as CAG and even GC (Xu et al., 2018; Luo et al., 2019). BBR shows good efficacy in the treatment of many diseases. Studies have shown that BBR can inhibit inflammation in hepatic fibrosis by reducing the production of TGF-β1 (Eissa et al., 2018). Berberine may enhance the chemosensitivity of gastric cancer cells to cisplatin by inhibiting PI3K/Akt signal pathway (Kou et al., 2020). And it also shows its therapeutic effect on gastroesophageal reflux disease rats by reducing the levels of pro-inflammatory biomarkers such as IL-1β, IL-6 and TNF-α (Hesari et al., 2018). However, its effect and mechanism on CAG induced by MNNG are still uncertain.

In our study, the efficacy of BBR was first verified on GES-1 cells. CCK8 and high-content live-cell imaging assays results showed that BBR could significantly improve the decrease of viability and cell damage of GES-1 cells induced by MNNG co-culture. The *in vivo* experiment also showed that the morphology of gastric mucosal glandular epithelial cells in BBR group was more complete, the arrangement was more regular, and the infiltration of inflammatory cells was significantly less than that in MNNG group. G17 is a specific indicator for the diagnosis of CAG (Lahner et al., 2019). Inflammation is an important factor affecting gastric mucosal hyperplasia and carcinogenesis, among which the most characteristic cells include IL-8, TNF-α and IL-1β (Pimentel-Nunes et al., 2019). We observed that BBR could significantly inhibit the expression of G-17 and inflammatory factors IL-8, TNF- α, IL-6 and IL-1β in CAG rats. The above results suggest that BBR has a certain therapeutic effect on CAG induced by MNNG.

Then we explored the possible mechanism of BBR improving MNNG-induced CAG. TGF-β signal is closely related to various chronic gastrointestinal inflammation and the pathogenesis of cancer. Many studies have shown that the level of serum TGF-β in patients with gastritis is significantly increased (Hong et al., 2010; Shamsdin et al., 2015). The expression of TGF- β1 was also significantly increased in patients with GC (Pak et al., 2016). It was found that the expression of TGF-β was up-regulated in GKO knockout CAG mice and mediated the conversion of CAG to GC (Donnelly et al., 2014). PI3K is an important downstream signal molecule of TGF-β1, which participates in the regulation of cell division, differentiation and apoptosis (Du et al., 2017). TGF-β1 can activate TGF-β1/PI3K/Akt/mTOR signal pathway. Studies have shown that inflammatory factors such as pro-inflammatory cytokines TNF-α and IL-1β are significantly up-regulated and PI3K/Akt axis is activated in mice with gastric ulcer (Shen et al., 2017). Xie and Liu, (2018) found that the expression of p-Akt in clinical HP positive CAG patients was significantly higher than that HP negative, and cell experiments showed that PI3K/Akt pathway was activated. This is consistent with our results. The results of *in vitro* experiment showed that the expressions of TGF-β1 and PI3K were significantly up-regulated after MNNG treatment. After BBR intervention, this increase could be significantly reversed, which was consistent with the SB-431542 treatment. Similarly, our *in vivo* experimental results also showed that compared with MNNG group, the expression of TGF-β1 and PI3K mRNA and protein in gastric tissue of BBR group were significantly decreased, and the phosphorylation levels of Akt and mTOR were significantly inhibited. In addition, the expression of related signals on the TGF-β1/PI3K/Akt axis further support our results. It has been reported that up-regulation of PTEN expression and inhibition of PI3K/Akt pathway can promote cell death (Li et al., 2017). mTOR can negatively regulate the expression of LC3-II and Beclin-1. Inhibition of PI3K/Akt/mTOR axis and activation of LC3-II, Beclin-1 play an important role in inducing human gastric cancer cell death (Lee et al., 2018). Our results showed that after the intervention of BBR, the expression levels of PTEN, LC3-II and Beclin-1 were significantly up-regulated, while the expression of P70S6k was significantly decreased. These results suggest that BBR may have a therapeutic effect on CAG through TGF-β1/PI3K/Akt axis.

There are some limitations in this study. There is no further discussion on the specific mode of cell death and autophagy in CAG. In addition to activating the PI3K/Akt signal pathway, whether TGF-β activates other signal axes at the same time plays a role in the occurrence and development of CAG. All these need to be further investigated.

## Conclusion

**FIGURE 9:**
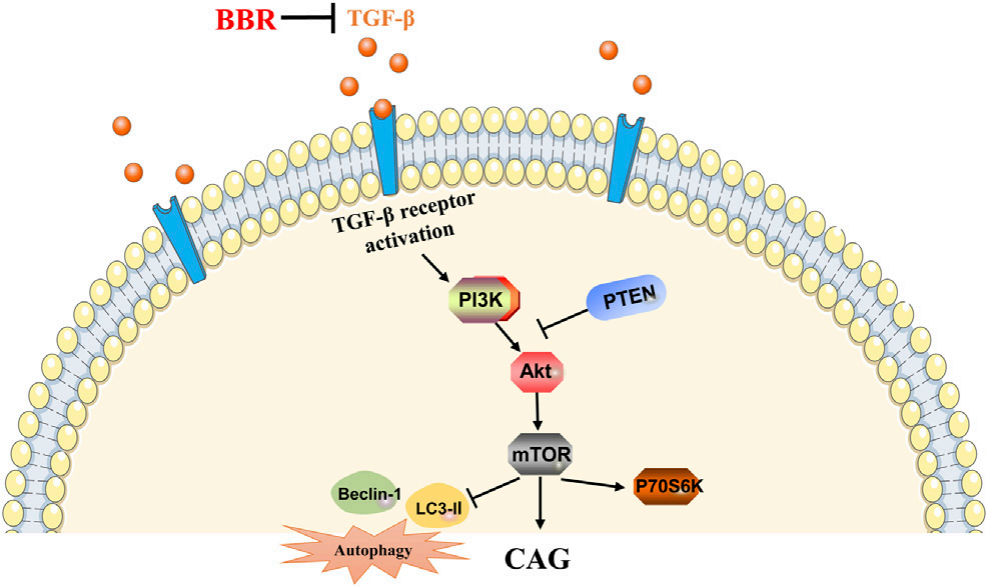
Mechanism of BBR in improving CAG induced by MNNG. →, activate; ┤, inhibit.

All in all, the results of this study suggest that BBR has a therapeutic effect on CAG induced by MNNG, and it may be closely related to TGF-β1/PI3K/Akt signal pathway (Figure 9). This study laid a theoretical foundation for the study of the pharmacological effects of BBR in the treatment of human CAG.

## Data Availability

The original contributions presented in the study are included in the article/Supplementary Material, further inquiries can be directed to the corresponding authors.
